# Developing a Clinical Program Based on the Needs of Patients With Chronic Lymphocytic Leukemia: Preparing for Illness Episodes

**Published:** 2017-07-01

**Authors:** Rose Bell

**Affiliations:** Department of Nursing, University of New York at Buffalo, Buffalo, New York

## Abstract

Patients with chronic lymphocytic leukemia (CLL) are part of a new category of survivors emerging in the population of Americans who are living with cancer. These survivors who have received treatment are living with an incurable cancer characterized by periods of exacerbations and remissions, yet little is known about this population’s needs from oncology providers. The purpose of this study was to identify the needs of the CLL survivor who has undergone at least one treatment for CLL. The results of this study led to the development of a clinical plan for these patients based on these needs. Grounded theory methodology was used to guide the study, which used semi-structured interviews to explore these patients’ unique needs. Twelve participants were recruited and asked to describe living their lives with CLL. The substantive theory from this study was the overarching category of "Living Under a Cloud of Illness." Additional primary categories included "In the Beginning," "Lens of Life," "Preparing for Illness," and "Advocating for Us." The results of this study can guide the advanced practitioner into patient-centered care based on the needs of the patient with CLL.

Despite advances in treatment, chronic lymphocytic leukemia (CLL) is a chronic disease that is still considered an inherently incurable hematologic cancer (Abrisqueta, Crespo, & Bosch, 2011; Beusterien et al., 2010; Molica, 2005; Stephens, Gramegna, Laskin, Botteman, & Pashos, 2005). Chronic lymphocytic leukemia is the most common form of leukemia, with 18,960 new cases diagnosed in 2016, and a type of cancer that starts in the lymphocytes or white blood cells within the bone marrow (American Cancer Society [ACS], 2016). As the disease progresses over time, it begins to invade the blood as well as other areas of the body such as the liver, spleen, and the lymph system. Invasion of these areas occurs as a result of abnormal accumulations of lymphocytes or white blood cells that lose their ability for programmed death, or the process by which a cell naturally dies. As a result, the abnormal cells accumulate at variable rates.

The symptoms experienced are related to the degree of tumor burden or the amount of disease present at any given time (Beusterien et al., 2010; Elphee, 2008). This tumor burden and subsequent disease progression can be demonstrated clinically by an increasing white blood cell count, lymph node enlargement, anemia, low platelet counts, enlargement of the spleen, and bone marrow involvement (Elphee, 2008; Evans, Ziebland, & Pettitt, 2012). Common symptoms of the disease include enlarged lymph nodes in the axilla, neck, stomach, and groin (Leukemia and Lymphoma Society, 2009). Other symptoms can include bleeding, bruising, weight loss, night sweats, and shortness of breath (Leukemia and Lymphoma Society, 2009). Some of the common causes of death in CLL are related to the progression of the disease and include bleeding, sequelae of anemia, and infections.

## EPIDEMIOLOGY, DISEASE COURSE, AND STAGING

Chronic lymphocytic leukemia is predominant in Western societies. It afflicts men twice as often as women and has an increased incidence in the elderly, with 75% over the age of 65 years (Abrisqueta et al., 2011; Beusterien et al., 2010). The majority of cases of CLL (70% to 80%) are discovered during a routine blood cell count, when patients are noted to have a higher-than-normal white blood cell count and are asymptomatic at the time of diagnosis (Abrisqueta et al., 2011; Beusterien et al., 2010).

The natural course of CLL initially begins in the indolent or slow phase in most cases, followed by disease progression (Evans et al., 2012). Chronic lymphocytic leukemia is hallmarked by the presence of clonal, mature B lymphocytes in the peripheral blood, spleen, bone marrow, and lymphoid tissue. The presence of these abnormal lymphocytes interferes with cell-mediated and humoral immunity and loss of immune memory. The course of CLL may vary widely, with about one-third experiencing a relatively indolent course and no treatment, one-third likely requiring treatment, and one-third requiring intensive treatment with more aggressive disease progression and perhaps death within a few years due to consequences of the disease (Elphee, 2008).

There are two primary staging systems for CLL: Rai and Binet. Staging is done to provide a universal language between providers and can be beneficial in determining individual prognosis and treatment. Aside from standard staging systems, there are also key indicators, including chromosomal abnormalities and markers, that can further assist in determining an individual’s prognosis and the aggressiveness of his or her disease.

## OVERVIEW OF TREATMENT AND SURVIVAL

Treatment of CLL is based on the severity of the disease and aimed at the control of disease and symptoms vs. cure. Frequently, those with CLL are not treated immediately, and instead implement a watchful waiting approach in which they are monitored regularly for symptoms and changes in blood cell counts. Among those who require treatment, patients who are frail or very elderly are more likely to be treated with a less toxic therapy (Leukemia and Lymphoma Society, 2009). Bone marrow transplant can be considered for a special subset of those with CLL. In general, transplant is reserved for those who are younger and have worsening disease or a more aggressive form of the disease.

The average median survival for patients with CLL has increased over time and is attributable to the development of treatments such as purine analog drugs, monoclonal antibodies, kinase inhibitors, and hematopoietic transplant. These interventions have subsequently resulted in an increase in 5- and 10-year relative survival rates (Brenner, Gondos, & Pulte, 2008; Cortes et al., 2010). Currently, the median survival of a patient who is diagnosed with CLL is approximately 10 years; however, prognosis is extremely variable (Abrisqueta et al., 2011).

Recent advances in medications include intravenous monoclonal antibodies, which originate identical immune cells that are clones of the parent cell, and radioimmunotherapies, which include antibodies labeled with a radionuclide and deliver radiation to the cells. These therapies have extended overall survival and resulted in the necessity for health professionals to better understand the needs of this population.

## SURVIVORSHIP

Advancements in CLL treatment have led to a growing population of survivors who are now living with this chronic, incurable form of cancer. These patients are diagnosed with a cancer that has an increasing life expectancy characterized by periods of exacerbation and remissions (Miller, K., Merry, & Miller, J., 2008). Chronic lymphocytic leukemia is considered a chronic cancer and despite advances in treatment, remains an inherently incurable hematologic malignancy (Abrisqueta et al., 2011; Beusterien et al., 2010; Molica, 2005; Stephens et al., 2005).

In one study addressing the unmet needs in the cancer survivor by Hodgkinson and colleagues (2006), the highest ranked need was having the best accessible medical care. Within that category were good communication with patients and collaboration between the health-care team and other services involved in patient care. Additionally, patients identified the need for ongoing, up-to-date, and understandable information for both themselves and others. They also ranked reduction of life stressors as an important need for survivorship. Other life needs cited in the literature have referenced relational, sexual, and vocational needs in those who have completed treatment for cancer.

However, these studies have been limited to those who had a reasonable expectation to be free of disease at the completion of treatment. Traditionally, most of the survivor studies have excluded those who have residual disease, incurable cancer, or recurrence. Historically, a survivor’s life needs to reflect a combination of personal factors such as relationships, coping skills, and situational factors such as social relationships and living circumstances (Thewes, Butow, Girgis, & Pendlebury, 2003). These factors are also affected by an individual’s ability to learn and understand needs that change over time and vary from person to person (Skalla, Bakitas, Furstenberg, Ahles, & Henderson, 2004; van der Molen, 2000). Age, culture, finances, education, fatigue, and presence of a consistent partner or relationship also influence the needs of the cancer survivor.

Evans et al. (2012) conducted a qualitative study that invited 37 patients with CLL to tell their stories. Each story was followed by a semistructured interview, with questions aimed at topics of interest in the narrative. Themes that emerged from the study included the difficulties patients experienced in coming to terms with the diagnosis, angst regarding the recommendations for watchful waiting vs. active treatment, a lack of information on the disease, feelings of being invisible to providers, and dealing with the disease symptoms (Evans et al., 2012). The study suggests that further emphasis be placed on the needs of this group and on communication and emotional as well as professional support. Further research is still needed to understand this unique group of cancer survivors, their concepts of survivorship, their needs, and the quality of their lives (Stephens et al., 2005).

## METHODS

A qualitative study using grounded theory methodology and participant interviews was conducted at a local oncology practice consisting of five offices. The purpose was to identify the needs of patients surviving with CLL and to develop a clinic program based on their needs. The study was conducted using one interview per participant. Saturation of data was reached after 12 interviews, and thematic saturation was achieved.

Eligible candidates spoke English, were at least age 18, and had a diagnosis of CLL for at least 1 year. Criteria for inclusion also included having received prior therapy (at least one treatment) for their diagnosis. The clinic population sampled was from various educational and socioeconomic backgrounds. Ethnic backgrounds included but were not limited to persons of Caucasian, Russian, and Norwegian descent. The subjects’ mean age was 68.25 years, with a range from 55 to 88 years old. Gender was primarily male (n = 10), and all were Caucasian.

## RESULTS

**Figure 1:**
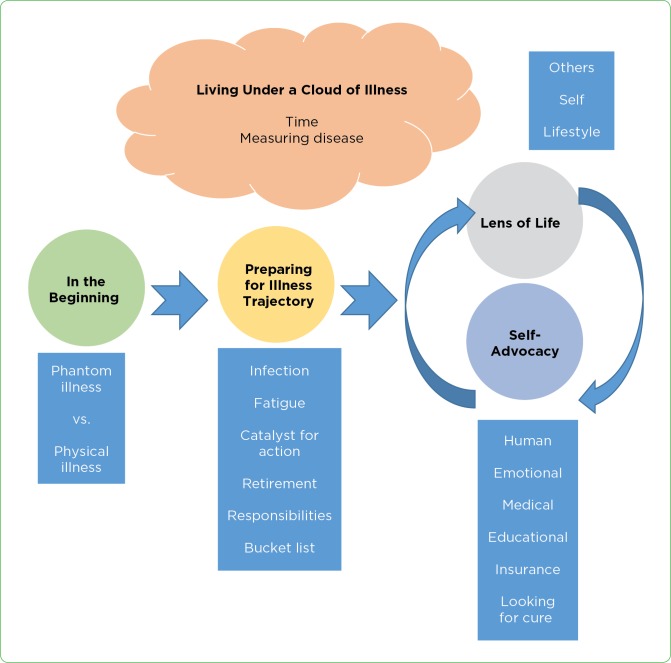
Surviving with CLL model for preparing for illness episodes.

Results of the study demonstrated an overarching category of "Living Under a Cloud of Illness." Additional primary categories include "In the Beginning," "Preparing for Illness Trajectory," "Lens of Life," and "Self-Advocacy" (Figure 1).

**Period of Initial Diagnosis**

In the beginning, the period after initial diagnosis was described as overwhelming: "My head was swimming," or "panic city." The initial diagnosis was divided into two distinct groups within those newly diagnosed: those with phantom illness and those who presented secondary to symptomatic disease. Those who presented with symptoms reported increased anxiety and confusion over ambiguous symptoms: "I was really sick and didn’t know why." Once the diagnosis was made, they reported a sense of relief at having a name for their symptoms.

The period of initial diagnosis for those who presented with symptoms was marked by feelings of fatalism but yet quite positive toward initiating treatment to alleviate their symptoms. These patients became less anxious as time passed. "As it went on and on, then I thought, oh, maybe I can beat this."

For those who were asymptomatic at the time of diagnosis, many reported the diagnosis being picked up during a routine physical exam. They reported subsequent feelings of disbelief and initially began to dwell on illness: "I had a tendency to let any symptoms upset me." The initial referral to an oncologist was also identified as a very stressful event and a catalyst to review their lives. 

**Focus After Diagnosis**

Both the symptomatic and asymptomatic groups described the period after diagnosis as "kind of a fog," and both groups reported relying on family or friends to listen and relay the information given early in the diagnosis.

After diagnosis, the participants reported a change in their outlook toward others and themselves (Lens of Life): "You look at life differently." In terms of describing themselves, several noted the following: an increased tolerance ("I’m more tolerant of people around me, especially family"); a new appreciation for life ("It has brought a new awareness and sensitivity that I might not have had"); and more empathy and patience in themselves and others. Additionally, they reported having a fuller life as a result of the diagnosis and a belief that "life is too short to be unhappy."

Although there is a general acceptance that life will change as a result of the diagnosis, there was a fear of changes in physical appearance as a result of CLL. Changes in appearance appeared to correlate with a transition from phantom illness to outward signs of a cancer diagnosis. This was particularly true in regard to hair loss: "First thing I said...is my hair or beard going to fall out? Well, that is the only thing that concerns me." Other outward signs of disease were observed by the participants and used as a gauge to determine whether they would proceed with future treatment.

*Changes in Relationships*: Most of the participants described changes in their relationships or view of others after diagnosis. Several reported a better relationship with their spouses since diagnosis. Others used the diagnosis as the primary motivation for change and ultimatums in relationships: "I said either you change or I’m out of here. And my husband changed dramatically, and our marriage got so much better, and that was really because of cancer."

Within the CLL population, there was a constant comparison with other acquaintances, friends, or family members who also had a diagnosis of CLL. The primary focus of their discussion centered on their blood cell counts, symptoms, and treatment and how they were doing comparatively and whether they were being treated in the same way.

Several of those interviewed reported both positive and negative changes in relationships and how they were treated by others after disclosure of their diagnosis. This was also affected by the amount of personal disclosure. Disclosure ranged from openly sharing information to limiting the circle of disclosure ("I almost don’t tell them because it doesn’t affect me"); or avoiding disclosure altogether ("I don’t want them to feel sorry for me"). Positive outcomes of disclosure to others included two benefits held to raise funds for the patient and his family.

One of the negative outcomes of disclosure occurred when a participant decided to run for a local association office. During the meeting, the participant reported that he heard another member state: "Why put him in? He’s going to die before long anyhow."

*Views on Survivorship*: In this group, views on survivorship were highly variable. At least two of those interviewed did not like the term "survivor" ("I would not use that word to describe myself") or did not like the label used to describe them personally ("I wouldn’t put it in the top 10 of who I am)." Survivorship was also described as day-to-day survival, and living with the diagnosis of CLL: "It’s always better on this side of the dirt than on the other side of the dirt."

There was a reluctance in the majority of patients to use the term survivor, as it was viewed as something earned: "I’ve not suffered; it cheapens others’ experiences." However, those who felt ill had more issues with control of their disease or were hospitalized at the time of diagnosis as a result of their disease, and appeared more likely to consider themselves survivors: "Absolutely, and it’s a raging battle because I am not in remission." Conversely, several patients described themselves only as survivors when the disease was controlled: "I think I am unless it should flare up again."

For many patients, a diagnosis of CLL resulted in reevaluation of their lifestyle and, in some instances, multiple life changes after diagnosis (e.g., diet, exercise, discontinuation of driving, avoidance of infection, and limiting social activities). The diagnosis was also a catalyst for more extreme life changes, including early retirement, contingencies for belongings, planning for others in their care, and fulfilling their bucket list.

*Diet*: The participants were split in their views on lifestyle changes after diagnosis. Those who have made changes in their diet and exercise did so with the belief that it helped them better control their disease: "I’m still keeping my blood count normal, but I do that by eliminating other things." A second group attempted to make dietary changes but experienced subsequent weight loss or gain directly related to their disease: "I had a slow weight reduction until I went through 6 months of chemo"; "I am not eating well because I have a lot of pain in my spleen." At the other extreme were those who thought they had been leading a healthy lifestyle prior to diagnosis and still developed cancer; hence, there was no need for changes.

*Illness Preparation*: With a diagnosis of CLL, there was an expectation of certain illness and preparation for illness as a result. Participants described this certainty of illness as "You know it is coming again," and along with the certainty is the readiness for treatment: "Let’s get it over with." Treatment is generally viewed as a means to return to normalcy. Initiating treatment was viewed positively in most of those who were symptomatic as a means to return to normalcy: "I was so sick for the first couple months that I basically just stayed in bed. Then I gradually got stronger and stronger." Once treatment is completed, they hopefully returned to their asymptomatic state again followed by the cloud: "You are in a holding pattern, and pretty soon you’re sick and then in limbo again," assessing and waiting for symptoms to occur; "There’s a little bit of dread you know, waiting for it to come around again to where I have to get chemo again sometime, but you just never know."

*Infections*: A common theme among the participants was a concern over their exposure to infections and the development of infections. Other changes in behavior after diagnosis included avoidance of social gatherings and crowds: "You have to err on the side of caution because of a compromised immune system. I can’t be around crowds"; "You become a germaphobe." Another participant described eating in a secluded area of the restaurant to avoid exposures: "I sit at a table away from everybody." One participant who had a history of sinus infections wore a mask during the summer months while doing yard work secondary to a fear of recurrent sinus infections.

*Fatigue*: Another common theme among participants was the impact of fatigue on their lives: "My energy has never really recovered; my memory was pretty off." Many reported the idea of budgeting activities within the confines of fatigue to adjust to their limitations: "I had to budget my energy, pace myself a little better, eat and drink a little better, a lot less alcohol or anything like that."

*Other Life Changes*: Participants described various other life changes as a result of their diagnosis, including retirement, a tentative plan for caregiver responsibilities, distribution of belongings, and development of a bucket list. At least two participants retired early as a result of being diagnosed with CLL: "I determined it wasn’t worth it, so I retired early." On reflection, one of the participants stated that the diagnosis of CLL precipitated the decision and that this was likely premature but prompted by an uncertain future: "I now wish I had worked a little bit longer."

Participants also described the development of a plan to distribute their belongings, and along with plans for their belongings, those interviewed described a contingency plan for caregiver responsibilities. Those who had caregiver responsibilities often described them as a catalyst for self-care behaviors focused on health promotion.

Lastly, participants described the need to live their lives to the fullest, and this need resulted in the development of a bucket list: "You better do all the things you want to do because you just don’t know if you’ll be in the hospital 2 weeks from now." Interestingly, the bucket-list items discussed during the interviews were not necessarily immediate future plans and involved long-term plans to include relocation: "I plan to purchase a motor home and move it down to New Mexico."

## TYPES OF RESPONSES

Responses regarding the needs of the patient with CLL from their caregivers were grouped into four categories. The patients described the need for emotional and educational support, their needs/expectations from the healthcare team, and insurance advocacy.

**Emotional Needs**

When describing what is needed from their medical professionals, the patients responded with the need for emotional support and the need for the professional to be emotionally present with them as they assimilate the diagnosis. This desire to be emotionally supported also extended into ancillary services. One of the participants underwent an ultrasound of a mass in the groin prior to diagnosis. The participant stated he became concerned when the technician’s demeanor changed. Additionally, one participant reported increased stress and more anxiety when the caregivers were speaking a different language while providing his care. This change provoked feelings of uncertainty and ongoing thoughts on whether there was something wrong with him or his care.

The desire for hope was also a common theme: "Treat me like I’m going to make it, and don’t act like I’m dying"; "I don’t want false hope, but I do want hope"; "You know we can beat this thing; just be positive I guess." Other characteristics described in this category included trust ("He’s like a wise father or something"), a calm approach, and a bedside manner that conveys hope.

There was a common expectation of expertise surrounding technically based procedures. They included intravenous skills and bone marrow biopsies: "If I was to say anything about a bone marrow test, though, we want to make sure it’s done by someone who’s done it before."

Participants also discussed the importance of honesty and approachability in their medical providers. Approachability was identified as feeling comfortable asking questions and receiving correct and honest information: "I can ask anything I feel I need to ask; I know you guys will give us correct answers to the best of your knowledge." Although there was a desire for honesty, they felt this information should again be delivered with the conveyance of hope: "Everybody in there (the infusion room) seems like they are pulling for you."

The other needs expressed were for timely follow-up from the provider and the office. Specifically, they wanted a quick turnaround time on reporting the results of blood work and imaging studies: "We didn’t get results; no phone calls back to let us know if they were OK or not."

**Educational Needs**

There was a wide variance in the desire for information. Some patients did a lot of research on their own after diagnosis, whereas others had family members who did the research on the disease. In other cases, more information was desired than was provided. Several times participants were given some information, but their understanding was limited secondary to the comprehension of medical terms: "So much jargon that it is just garbage"; "The majority of books are all these studies that you know are…just the vocabulary."

The last category included several patients who wanted limited to no information. This was related to feelings of being overwhelmed, more acute illness at the time of diagnosis, and the disease being incurable.

**Insurance Needs**

A major concern for several of the study participants was how to pay for treatments and navigate through a complex insurance process. This concern was exacerbated when they had disease progression that required treatment.

As new drugs are developed, treatment options continue to change. New drug recommendations are used with more frequency and are associated with higher costs to the patient: "How the hell am I going to get this paid?" This had led several providers to address insurance coverage and cost to the person up front before treatment began, yet this up-front discussion may result in further anxiety for the person with CLL.

**Living With Chronic Lymphocytic Leukemia**

Participants were asked to describe what it is like to live with CLL. After diagnosis, they reported feelings of overall life uncertainty: "A cloud kind of hangs over you; it is a big question mark on your life." And this uncertainty continues throughout their daily lives. Several of the patients used the word "cloud" to more fully describe this uncertainty:

- "It’s like living under a cloud."
- "There’s a cloud there, and it’s got your name on it."
- "It’s going to sweep in, and you’re going to go through the same thing you just went through a year before, the month before, or whatever."

For those interviewees who did not specifically use the word "cloud" to describe their lives since diagnosis, they described similar feelings of ongoing anticipation. Since the disease is considered incurable, all of the participants described the acceptance of living with CLL and incorporating the disease as well as learning to live with it: "I had to let go of it early on, realizing I can’t do anything about it. I just had to learn to live with it." Although they reported that they do not worry they are in any imminent danger of death (one patient stated, "I know I’m not going to die tomorrow or anything"), there is a constant worry of illness. During the periods where there is an absence of symptoms, worry about when the disease will recur remains: "It’s always in the back of my mind. What does this mean for me long term? Is it going to be my demise?"

The uncertainty of the disease course often results in the ongoing personal conundrum in assessing a reasonable life expectancy. "My current concerns are, you know, is it 5 years, 10 years, 20 years?" "What does ’long term’ mean?" This prognostic time frame and the focus on survival rates can sometimes result in more fear for the future.

The participants sought to measure life expectancy through symptoms of disease progression such as complete blood cell counts and more specifically, white blood cell counts: "Waiting for results, and you don’t know what you have, 1 week, 1 month, or 5 years." Blood cell counts are often patients’ only objective symptom for monitoring their disease: "I have not had any symptoms except the high white blood cell count." Therefore, these counts are watched very carefully by patients as a measurement of wellness, disease recurrence, and life expectancy.

**Other Concerns**

There were other concerns reported. There was a common interest among patients with CLL about the etiology of their disease. For military veterans who served during the Vietnam era, Agent Orange was raised as a potential contributing factor vs. cause of their disease ("One of the spin-offs to exposure to herbicides is CLL"). Additional beliefs about etiology included heredity ("Having too many white blood cells that are bad is what actually starts it, but what actually causes it? Is it hereditary or environmental?") and exposure to chemicals ("Well, I heard it can be caused from... the preservatives they use in food").

There was also interest in the cause of death for someone with the diagnosis of CLL. Although several of the patients stated that they didn’t really want to know and had never asked, they had concluded that since their risk for infections was increased, infection was likely the cause of death. This was especially true for those who had been treated for infections in the past: "It’s like you don’t die of CLL; you die of complications of CLL."

Various daily activities or intermittent measurements were frequently described to self-monitor the activity of their disease. They included routine evaluation of enlarged lymph nodes ("I did have little lumps under my armpits; that’s the only thing I had"); while shaving ("The only time I think about it is in the morning when I’m shaving; I always check to see if I am swollen"); and evaluation of complete blood cell counts during their visits and other symptoms such as sweating ("Is it menopause or CLL?").

One of the most interesting themes from this study revolved around the idea of cure in CLL. There was a common thread of hope for cure. One participant described the idea of cure as always present but not necessarily the focus of those providing their care: "The doctor you go to isn’t looking for a cure. Cure isn’t where his mind is; cure is where your mind is." Patients also expressed interest in clinical trials at cancer centers as a path for cures in the future. Several participants described continuing with current treatment and maintaining stable disease in the hope of future improvement in therapies or a cure.

## DISCUSSION AND IMPLICATIONS FOR THE ADVANCED PRACTITIONER

The results of this study have laid the groundwork for the development of survivor education, plans, and clinical pathways (Table) in CLL and a further understanding of those who have received treatment for CLL. Comprehension of what it means to have the initial diagnosis and treatment followed by a life built under a cloud of disease exacerbations, remissions, and preparation for illness provides caregivers, nurses, and providers with a richer understanding of the emotional, physical, and social issues that surround a diagnosis of CLL. Within the identification of two categories of newly diagnosed patients with and without symptoms, there is further understanding into the quality-of-life differences, patient motivations, and educational needs in both groups.

**Table 1:**
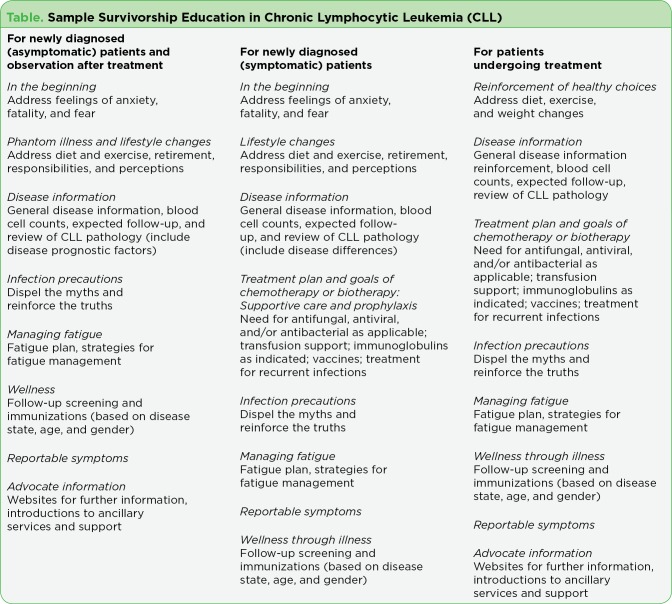
Sample Survivorship Education in Chronic Lymphocytic Leukemia (CLL)

Theories used to address survivors acknowledge their needs and a belief that these needs change along a life continuum (Mullan, 1985; Thewes et al., 2003; Vivar & McQueen, 2005). The participants with CLL who received at least one treatment for their disease all developed strategies to better cope with the exacerbation and remission cycles of their disease.

**Figure 2:**
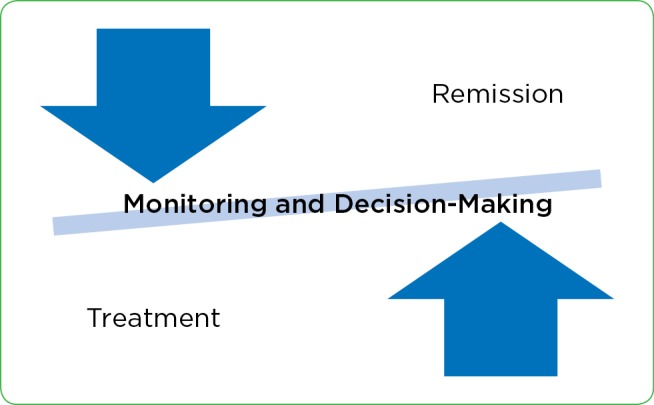
Living life under a cloud of remission and exacerbations in chronic lymphocytic leukemia.

The participants also developed life arrangements and priorities that focused on a life lived under a cloud of remission with a plan for illness exacerbations (Figure 2). A critical understanding of the decisions that are likely to be considered after a diagnosis of CLL and the preparation for illness is crucial in providing quality care to survivors. Many issues, including the development of the bucket list, consideration of retirement, care of others, lifestyle changes, and designation of belongings, emerge from the diagnosis. Health-care professionals should be aware of the changes being considered to serve as informed navigators through the patient’s decision process.

**Providing Education**

The concept of fog after diagnosis as well as decreased memory with fatigue guides us toward inclusion of the family in the education provided as well as the plan of care. The plan of care should include careful yet honest discussion of time surrounding the diagnosis, with emphasis on hope throughout the phases of the disease. Prognosis with time appears to be an important catalyst for future planning. Vague reassurance was viewed negatively, and most participants sought the information from other sources (Shanafelt et al., 2009). This challenges the question of how much information is too much and provides the basis for further investigation.

Cancer patients require information about their disease and its treatments to provide them with an understanding of what to anticipate in the treatment of their disease. Such information includes treatment side effects and possible complications to assist in preparing them for a "change in lifestyle and uncertainty that lies within their diagnosis" (McCaughan & Thompson, 2000, p 852). In a study conducted with patients in the acute treatment phase, Skalla et al. (2004) identified themes about the treatment process, educational needs, sources of reliable patient information, side effects, and expectations.

Educational measures identified from this study included infection precautions, dietary measures, use of alternative and complementary therapies, and side effects of medications (including physical changes). Information should be simple and communicated in plain, understandable language.

Treatment expectations are of particular importance, since the "most distressing side effects reported were the ones the patient was least prepared to experience" (Skalla et al., 2004, p 317). Treatment knowledge is vital to self-care activities and coping (Adams, 1991; McCaughan & Thompson, 2000). Patients reportedly learned primarily from infusion nurses, spouses, and other patients (Skalla et al., 2004). Of particular note was that patients who received information from other cancer patients were more positive toward their treatment (McCaughan & Thompson, 2000).

Studies also reported that patients wanted all the information available, but that obstacles existed in obtaining that information (McCaughan & Thompson, 2000; Skalla et al., 2004). Health-care provider obstacles have been reported from survivors as a barrier to obtaining information about their cancer therapy. These obstacles included lack of access to providers, providers’ reported lack of knowledge or sensitivity to the life needs of cancer patients, and providers not wanting to answer questions (Skalla et al., 2004).

Communication should be aimed at both the patient and the family members as part of a whole unit, both pretreatment and posttreatment. This is particularly important early in the diagnosis, when the patient may not fully hear or understand what is being discussed.

Many of the survivors who received vague reassurance from their medical team relied on the word of friends, acquaintances, or the Internet for information. This resulted in many misconceptions regarding their disease, etiology, prognosis, and treatment. Education should be provided in simple and direct terms to both survivors and caregivers. It can be either written or verbal and should include an understanding of blood values, infection precautions and prevention, and fatigue management. Effective symptom management for better overall quality of life cannot be overemphasized. Since many patients present without symptoms, their understanding of the watchful-waiting treatment approach is imperative to decrease anxiety and fear regarding their disease. Later, if they should require treatment, discussion of the treatment and side effects should be included in the plan.

Health-care professionals should understand the significance of prognostic time, symptom discovery, and timely delivery of test results to patients with CLL. Health-care workers should be sensitive to the use of other languages in the presence of patients and of gestures used, such as whispering. These actions may result in increased anxiety and a fear that something is wrong.

**Meeting Emotional Needs**

Throughout the interviews, patients continued to emphasize the need for honesty, approachability, and establishing trust. They also wanted an honest method that was infused with hope. Some described it as a kind bedside manner. Others stated they did not want to be treated as if they were dying of cancer but rather living with cancer.

Today, the ongoing evolution of technology in medicine can lend itself toward a technologic vs. humanistic approach to patients. Each patient in the study tried to describe their need for and the importance of an emotional presence from the physician and nurses as well as from ancillary services such as radiology professionals. The incorporation of empathy and a positive outlook from the health-care team was a key factor in their overall well-being.

The advanced practitioner should understand the concept of what living under a cloud means for the person with CLL and incorporate it into the care plan. We need to understand the utility of the comparative partner, which is often chosen for side-by-side comparison of the disease to clarify misconceptions and identify differences within the same disease spectrum. Additionally, we need to become familiar with the changes that may occur in the patients’ view of themselves, others, and their lifestyle as a result of their diagnosis. Staff can be instrumental in assisting these patients with healthy lifestyle changes, such as diet and exercise, as well as symptom management.

**Developing Survivorship Care Plans**

The Table illustrates an education plan for CLL survivors. The development of plans for patients with incurable, chronic cancers should be considered. The Commission on Cancer’s standards have been initiated for the development of survivorship plans in the posttreatment population. The purpose was to develop and deliver a care summary to patients as well as to other providers involved in their care.

The standards for the plans include the following: preparation by providers who coordinate oncology treatment; a record of care received including disease characteristics; and a written follow-up plan given to patients on completion of treatment (American College of Surgeons, 2012; Ferrell, Virani, Smith, & Juarez, 2003; National Coalition for Cancer Survivors, 2016). Given the chronicity of CLL and the likelihood for multiple treatments, a survivorship summary should include a record of care as well as written follow-up, recognition of acute and late effects, and symptom management. It may be considered a continuity and educational tool for this disease and other similar types of chronic cancers.

Routine screening in this group may not be warranted or indicated, depending on the patient’s age at diagnosis and current federal guidelines.

**Addressing Financial Concerns**

Finally, the financial impact of CLL treatment can be devastating for patients and their families. The rising cost of drugs, the decreases in Medicare coverage, and issues with veterans’ benefits are ongoing concerns for many patients undergoing treatment for CLL. Advocates for financial assistance programs and advisors to assist with financial aid are necessary for this patient population.
